# Effect of chloroquine on reducing HIV-1 replication *in vitro *and the DC-SIGN mediated transfer of virus to CD4^+ ^T-lymphocytes

**DOI:** 10.1186/1742-4690-4-6

**Published:** 2007-01-30

**Authors:** Marloes A Naarding, Elly Baan, Georgios Pollakis, William A Paxton

**Affiliations:** 1 Laboratory of Experimental Virology, Department of Medical Microbiology, Center for Infection and Immunity Amsterdam (CINIMA), Academic Medical Center, University of Amsterdam, Meibergdreef 15, 1105 AZ Amsterdam, The Netherlands

## Abstract

**Background:**

Chloroquine (CQ) has been shown to inhibit HIV-1 replication *in vitro *as well as *in vivo *and has been proposed to alter the glycosylation pattern of the gp120 envelope. These activities indicate that the compound can be used not only as an effective HIV-1 therapeutic agent but also as a modulator of the gp120 envelope protein structure enabling for the production of broader neutralizing Ab responses.

**Results:**

We confirm here that HIV-1 replication on CD4^+ ^T-lymphocytes can be reduced in the presence of CQ and show that the reduced replication is producer cell mediated, with viruses generated in the presence of CQ not being inhibited for subsequent infectivity and replication. By analysing the gp120 envelope protein sequences from viruses cultured long-term in the absence or presence of CQ we demonstrate variant evolution patterns. One noticeable change is the reduction in the number of potential N-linked glycosylation sites in the V3 region as well as within the 2G12 Ab binding and neutralization epitope. We also demonstrate that HIV-1 produced in the presence of CQ has a reduced capacity for transfer by Raji-DC-SIGN cells to CD4^+ ^T-lymphocytes, indicating another means whereby virus transmission or replication may be reduced *in vivo*.

**Conclusion:**

These results indicate that CQ should be considered as an HIV-1 therapeutic agent with its influence exerted through a number of mechanisms *in vivo*, including modulation of the gp120 structure.

## Background

The anti-malarial drug chloroquine (CQ) and its hydroxyl analogue hydroxychloroquine (HCQ) have both been shown to inhibit the *in vitro *replication of HIV-1 and HIV-2 [1]. The cheap cost and wide-availability in resource restricted settings make them prime candidates as antiretroviral agents, most likely to be used in conjunction with other anti-HIV-1 medications. A previous report has indicated that CQ may mediate its effect through modulating glycosylation patterns of the HIV-1 gp120 envelope protein [2]. Since HIV-1 neutralizing Ab responses can be modulated by alterations in the potential N-linked glycosylation (PNG) sites of gp120 [3-5], CQ and HCQ may therefore have the beneficial effect of changing the immunogenicity of the molecule and induce a broader Ab response.

The HIV-1 inhibitory effect of CQ and HCQ is likely mediated by variant properties of the drugs. As a weak base CQ is known to increase pH in lysosomal and trans-Golgi network vesicles [6], thereby disrupting the cellular acid hydrolase enzymes and altering the level of post-translational modification of newly synthesized proteins and reducing the level of gp120 glycosylation. The cellular endosomal pH has also been shown to be increased through CQ treatment which can lower IL-6 synthesis [7]. Down-modulation of IL-6 has been shown to diminish HIV-1 production from chronically infected T-cells and monocyte cell-lines [8], providing an additional HIV-1 suppressing effect. CQ has also been shown to decrease Tat-mediated transactivation of the HIV-1 LTR *in vitro*, thereby decreasing HIV-1 production [9].

Dendritic cells (DCs) have been implicated to play an important role in the transmission of HIV-1 and the establishment of infection through capturing virus and enhancing infection of CD4^+ ^T-lymphocytes [10-12]. DC-SIGN has been shown to specifically interact with HIV-1 and allow for the enhancement to infection [13-15], although an array of C-type lectins have been postulated to perform the same function [16,17]. The interaction of HIV-1 with DC-SIGN can lead to either infection of DCs or internalization of the virus and subsequent transfer [18,19]. The interaction of HIV-1 and DC-SIGN is mainly dependent on the glycosylation of gp120 and in particular the V3 region of the protein [20].

Several clinical trials have been performed where CQ or HCQ was given to HIV-1 infected individuals. In one study a decrease in HIV-1 viral load measurements was observed [21] whilst in another a decrease in plasma CA-p24 levels was noted in comparison to the control group [22]. No alterations to CD4^+ ^T-lymphocyte counts were identified in either study. In one trial a decrease in IL-6 and immunoglobulin G levels were found, suggesting a further means whereby HIV-1 viral loads can be modulated [22].

## Results

### Inhibition of HIV-1 replication by CQ

**Figure 1:**
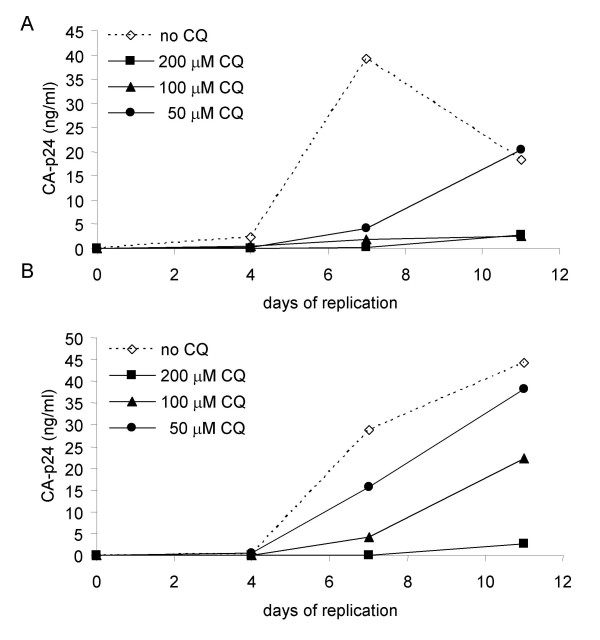
**Viral replication in the presence of CQ**. A) JR-CSF (R5) virus B) LAI (X4) virus replication was monitored in the presence of 200 μM, 100 μM, 50 μM of CQ or in the absence of CQ. Viral input for the replication assay was 100 TCID_50_/ml with the CA-p24 concentration determined during the course of the infection.

To confirm that CQ has an inhibitory effect on the *in vitro *replication of HIV-1 we separately cultured an R5 (JR-CSF) and X4 (LAI) virus on CD4^+ ^T-lymphocytes and monitored replication in the presence of variant concentrations of CQ (200, 100 and 50 μM). We observe that CQ inhibits the replication profile of both viruses in comparison to the control cells (Fig. 1). When comparing the dose dependent inhibitory effect of CQ on viral replication the R5 virus (Fig. 1A) appears more sensitive than the X4 virus (Fig. 1B), suggesting a co-receptor phenotype restriction to inhibition by CQ. The observed inhibition by CQ was not due to enhanced cell death since cell counts and viabilities were identical in the 100 and 200 μM CQ cultures to the non-CQ treated control cells during one week of culture (data not shown).

**Figure 2:**
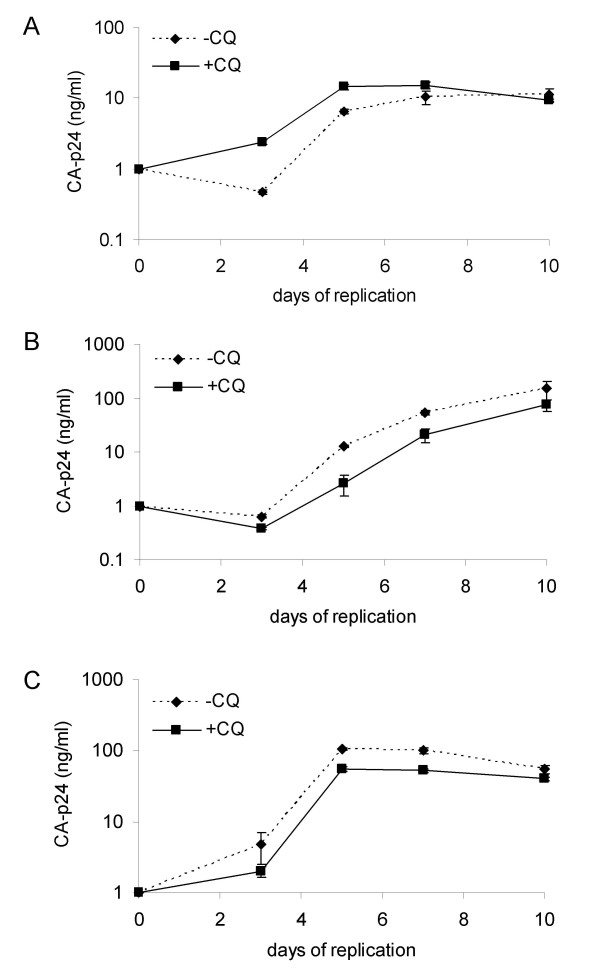
**Viral replication of C33A produced viruses in the presence of CQ**. A) JR-CSF (R5) replication, B) 299.10 (R5X4) replication and C) LAI (X4) replication. All three viruses were produced by transfection of C33A cells pre-treated with 100 μM CQ or in its absence as a control. The replication capacity of the produced viruses were determined on CD4^+ ^T-lymphocytes in the absence of CQ. CA-p24 at 1 ng/ml was used as viral input with the CA-p24 concentration determined during the course of the infection. Standard deviations are depicted in all panels. All replications were performed in triplicate.

In order to determine whether the inhibitory effect of CQ was mediated through altered infectivity of generated virus particles we analyzed replication on CD4^+ ^T-lymphocytes of HIV-1 produced in C33A cells pre-treated with 100 μM CQ. The viruses JR-CSF (R5), 299.10 (R5/X4) and LAI (X4) produced from cells not treated with CQ showed higher CA-p24 levels than viruses produced from cells treated with CQ (data not shown). When we studied the replication kinetics of the viruses away from CQ with a set CA-p24 viral input there was no difference in replication of the viruses generated in the presence or absence of CQ (Fig. 2). TCID_50_/ml values were identical for all three viruses generated in the presence or absence of CQ (data not shown). These results indicate that viruses produced in the presence of CQ are as equally infectious as those produced in its absence and that the effect of the drug on lowering CA-p24 production is mediated at the cellular level.

### Prolonged culture in the presence of CQ does not alter replication of HIV-1

**Figure 3:**
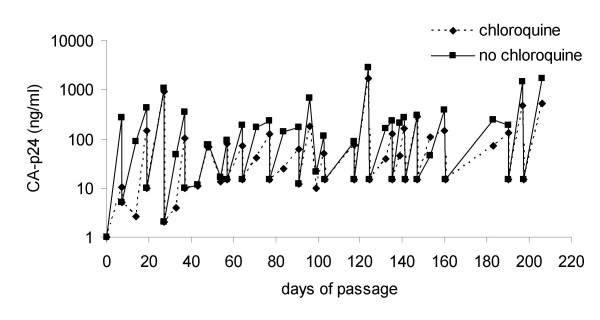
**Prolonged passage of HIV-1 in the presence of CQ**. An R5X4 virus (293.10) was cultured for 206 days in the presence or absence of CQ (100 μM). The concentration of CA-p24 was determined in culture supernatants on either day 7 or 10 of culture and 15 ng/ml CA-p24 was added to fresh CD4^+ ^T-lymphocytes. The culture was monitored for CA-p24 and the culture in the absence of CQ is depicted with a solid line and the culture in the presence of CQ is depicted as a broken line.

To monitor the effect of long-term culturing of HIV-1 in the presence of CQ we passaged virus 293.10 (R5X4) for 30 weeks either in the absence or presence of CQ (100 μM). Each week, or when CA-p24 levels were sufficiently high, a set viral load (15 ng/ml) was transferred to fresh CD4^+ ^T-lymphocytes and cultured. CA-p24 production was consistently lower for the virus passaged in the presence of CQ compared to the control passsaged virus (Fig. 3), even after 206 days of culture (30 passages). These results demonstrate that HIV-1 does not evolve to escape the inhibitory effects of CQ.

**Figure 4:**
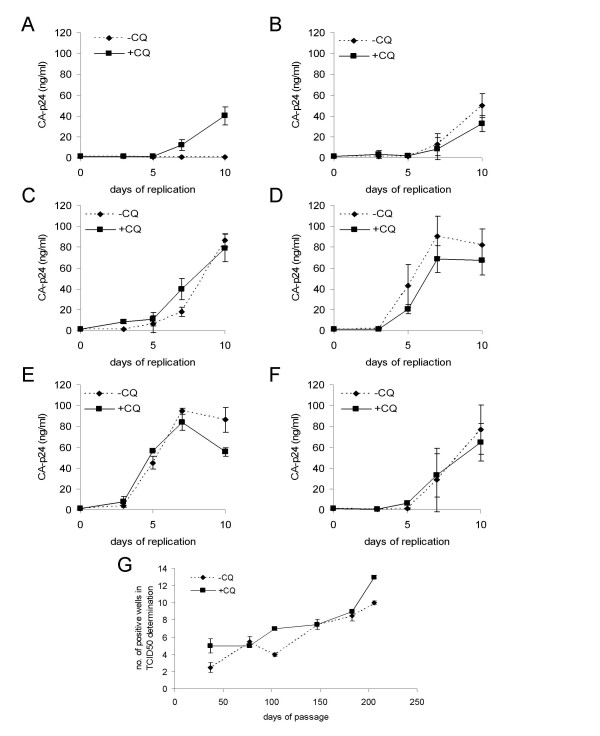
**Replication of CQ passaged virus**. The replication of CQ passaged or the control passaged 293.10 viruses were tested for their replication in the absence of CQ. CA-p24 or 1 ng/ml was used as input for monitoring replication A) day 37 of passage, B) day 77 of passage, C) day 103 of passage, D) day 147 of passage, E) day 183 of passage and F) day 206 of passage. Standard deviations are depict in all panels. All virus replications were performed in triplicate. G) Determination of TCID_50_/ml values of passaged viruses in the absence or presence of CQ. Viral infectivity of the viruses passaged in the absence or presence of CQ (days 37, 77, 103, 147, 183 and 206) was measured on CD4^+ ^T-lymphocytes. Standard deviations are depicted.

Since we have shown previously that viral replication was not altered after HIV-1 production on C33A cells in the presence of CQ we wished to identify whether this was the same for the long-term cultured virus stocks. The replication profile of harvested viruses from various time-points during the passage in the presence or absence of CQ was determined on CD4^+^T-lymphocytes (Fig. 4). The replication at day 37 showed an increase in replication for the CQ passaged virus population versus the non-CQ treated culture (Fig. 4A). On the contrary for day 77 (Fig. 4B), day 103 (Fig. 4C), day 147 (Fig. 4D), day 183 (Fig. 4E) and day 206 (Fig. 4F) no differences in replication between the CQ passaged viruses and the non-CQ passaged viruses were observed. These results indicate that there is no difference in replication of HIV-1 after long-term culture in the presence or absence of CQ, although the virus from the CQ treated day 37 culture showed an enhanced replication over the non-CQ treated stock. The fact that the later viruses did not show such an increase indicates that the result observed for the day 37 CQ passaged virus was most likely due to experimental variation and reflects the poor infectivity of the viruses from that time-point. However, the main finding is that CQ did not diminish the replication capacity of HIV-1. TCID_50/ml _values were determined for stocks generated on days 37, 77, 103, 147, 183 and 206 during the prolonged passage in the absence or presence of CQ. Both culture conditions demonstrated an increased infectivity of virus over time (Fig. 4G), indicating that viruses in the presence of CQ adapt as efficiently as non-CQ treated cultures. This again reiterates that CQ exerts a cellular restriction to viral production and not a direct effect on viral infectivity.

### Sequence analysis of the viruses passaged in the presence of CQ

**Figure 5:**
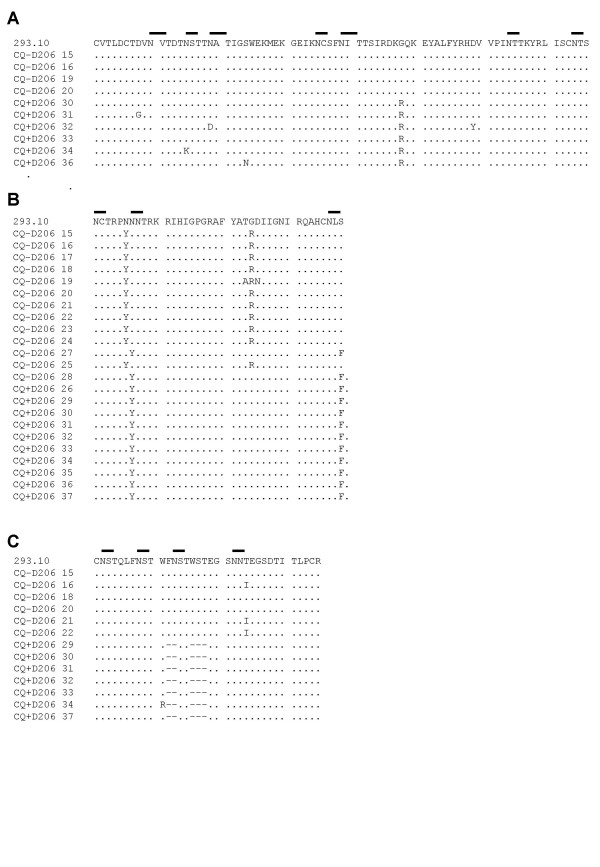
**Sequence analysis of passages viruses in the presence or absence of CQ**. HIV-1 RNA was isolated from culture supernatant and viral RNA was converted to cDNA and then subjected to a nested PCR in order to amplify a fragment covering the V1V2 – C4 region of the gp120 gene. Sequence analysis was performed on several clones of the CQ and control passages. The sequence of the original virus 293.10 is shown. A) the V1V2 region. B) the V3 region including the PNG site at the base of the loop. C) the V4 region. The black lines above the original sequence represent PNG sites.

A previous study has suggested that CQ can modify the PNG patterns of the gp120 envelope [2]. We therefore wished to determine whether HIV-1 passaged in the presence of CQ had a similar gp120 envelope sequence to virus passaged in the absence of CQ. DNA sequence analysis of a number of cloned PCR products of gp120 identified that the overall amino acid charge of the V1V2 region (Fig. 5A) is significantly higher for the CQ passaged virus compared to the control passage (P = 0.001), or the original virus (P = 0.001) (Table 1). On the contrary, the overall positive charge of the gp120 V3 region (Fig. 5B) is significantly lower (P < 0.0001) in the CQ passaged virus but equal to the original virus (Table 1). A significant decrease in V4 region length (Fig. 5C) is also identified in the CQ passaged virus in comparison to the control (P < 0.0001), or the original 293.10 virus (Table 1). Of particular interest is the observation that the PNG profile of the V3 region (Fig. 5B) was significantly reduced after passage of the 293.10 virus in the presence of CQ with the virus reducing the number of PNG sites in V3 region from 2 to 0 (P < 0.0001), whilst in the non-CQ treated culture it is reduced from 2 to 1.7 (Table 1). Overall the sequence analysis reveals that there are differences in the envelope sequences of viruses cultured in the presence of CQ that may have an influence on the virus phenotype or the immunogenic properties of gp120.

**Table 1:** Sequence comparison between the passage of 293.10 for 206 days with or without CQ

			**day 206 of passage**		
				
	**gp120 region**	**293.10**	**CQ (#)**	**Control (#)**	**P value**
	V1V2	0	1.33 (± 0.52)	0 (± 0)	0.001 *
Charge	V3	4	4 (± 0)	4.85 (± 0.36)	< 0.0001 *
	V4	-2	-2 (± 0)	-2 (± 0)	equal

	V1V2	76	76 (± 0)	76 (± 0)	equal
Length	V3	36	36 (± 0)	36 (± 0)	equal
	V4	28	23(± 0)	28 (± 0)	< 0.0001 *

no. of	V1V2	7	6.67 (± 0.52)	7 (± 0)	0.24
N-glycosylation	V3	2	0 (± 0)	1.69 (± 0.75)	< 0.0001 *
Sites	V4	3	2 (± 0)	2.5 (± 0.58)	0.09

charge, length and potential N-glycosylation sites were determined from the amino acid sequences

# Standard deviations

* P values are considered significant

### Prolonged passage of HIV-1 in the presence of CQ results in a loss of PNG sites important for 2G12 binding

We compared the gp120 sequences of the passaged viruses with what is known for the 2G12 binding site, a monoclonal Ab with broad neutralizing activity against HIV-1 subtype B isolates. This antibody has a known PNG component to its recognition epitope [23]. For the virus passaged in the presence of CQ we observed a loss of two PNG sites (332 and 397) that have been shown to express carbohydrates important for 2G12 binding [23], as well as an additional site in the V3 region of gp120 (data not shown). The PNG site expressing carbohydrates involved in 2G12 binding (397) is lost in the V4 region due to a deletion of 5 amino acids. Loss of a PNG site in the V4 region is also observed in the control passage (Table 1) but does not involve this specific site under question since the deletion is 7 amino acids upstream from position 397.

### DC-SIGN mediated transfer of HIV-1 is decreased for both C33A generated viruses and after prolonged culture in the presence of CQ

**Figure 6:**
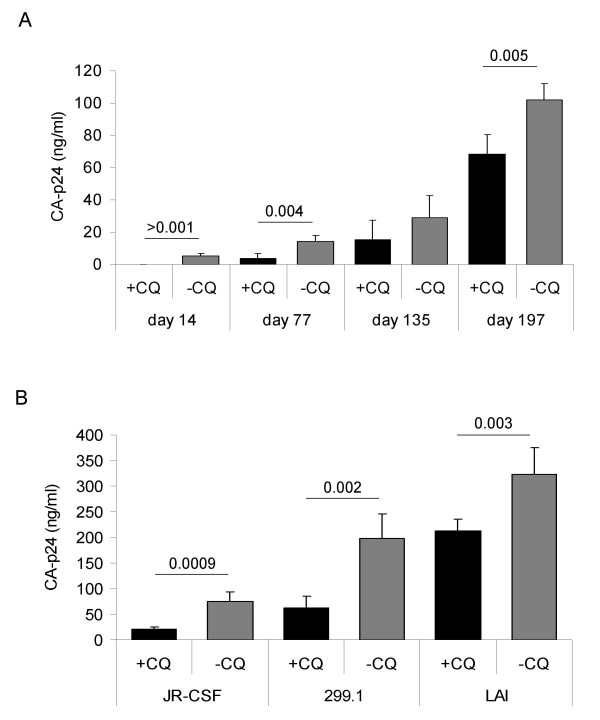
**DC-SIGN mediated transfer of CQ passaged viruses and C33A derived viruses in the presence of CQ**. Raji and Raji-DC-SIGN cells were incubated with viruses before washing with PBS and addition of CD4^+ ^T-lymphocytes. CA-p24 levels were determined at day 7 by standard ELISA. The CA-p24 levels of transfer by Raji cells alone were subtracted from the CA-p24 values of transfer observed with Raji-DC-SIGN cells. A) DC-SIGN dependent transfer of viruses cultured long-term in the presence or absence of CQ (days 14, 77 and 197). B) DC-SIGN dependent transfer of JR-CSF, 299.10 and LAI virus produced in C33A cells either in the presence or absence of CQ. Standard deviations are depicted in both panels and P-values given.

Since PNG sites were altered in the CQ passaged viruses and these events are known to be involved with HIV-1 binding to DC-SIGN [20,23] we tested the efficiency by which the viruses were transferred by Raji-DC-SIGN cells to CD4^+ ^T-lymphocytes. When comparing the viruses produced on days 14, 77, 135 and 197 we observe that for viruses produced in the absence of CQ there is a significantly higher level of DC-SIGN mediated transfer than viruses produced in the presence of CQ (day 14, P = 0.001; day 77, P = 0.004; day 197, P = 0.005) (Fig. 6A). These results indicate that the alteration of the PNG sequence of gp120 may alter its binding to DC-SIGN, or alternatively the glycosylation machinery of the cell can influence the interaction of the virus with DC-SIGN. To test the latter we monitored the Raji-DC-SIGN mediated transfer to CD4^+ ^T-lymphocytes of viruses produced on C33A cells (CQ or non-CQ treated). All three viruses were shown to have a reduced capacity for DC-SIGN mediated transfer when produced in cells treated with CQ over viruses generated in non-CQ treated cells (JR-CSF, P = 0.0009; 299.10, P = 0.002; LAI, P = 0.003) (Fig. 6B). This result indicates that the same virus produced in the presence of CQ has a reduced capacity for transfer by DC-SIGN expressing cells to CD4^+ ^T-lymphocytes.

## Discussion

We demonstrate, in support of previous *in vitro *and *in vivo *studies [1,21,22,24-27], that CQ has an inhibitory effect on HIV-1 production. We further demonstrate that viruses produced in C33A cells or which have been extensively passaged through CD4^+ ^T-lymphocytes in the presence or absence of CQ show no difference in their infectivity profile and TCID_50_/ml values when cultured away from CQ, indicating that the inhibitory effect on viral replication is provided at the level of the producer cell. Sequence analysis of the viruses after prolonged passage in the presence or absence of CQ demonstrates a loss of PNG sites in the gp120 region. Previous results have shown that N-glycosylation is of importance for the pathogenisis of HIV-1 but does not alter replication or infection of target cells [28], which is in correspondence to our results. CQ has been shown previously to reduce viral yield *in vitro *[1,25,27], but also viral infectivity [1,27]. However, in our study we do not observe inhibition of infection of CD4^+ ^T-lymphocytes. This may be explained by the fact that in our experiments we compensate for the presence of CQ in the produced virus stock, thereby eliminating the possibility of CQ transfer inhibiting viral replication in the stocks produced in the presence of the drug. It also should be noted that the inhibitions observed in the two previous reports are low, varying between 5 – 50% of viral inhibition.

Sequence analysis of HIV-1 extensively passaged through CD4^+ ^T-lymphocytes revealed a number of genotypic differences between the CQ and the control passaged virus, including an increased V1V2 charge, a lack of increase in the V3 overall charge, a shortened V4 region and modulation of the PNG patterns in the variable loops, suggesting some pressure on the envelope structure exerted through culturing in the presence of CQ. Interestingly, it has been reported that CQ may modulate the PNG sites of the gp120 envelope [1,2,27], which is supported by our results. When we specifically analyze the epitope of the 2G12 neutralizing Ab, which is known to be expressed by PNG sites [23], we observe a high degree of variation with a number of PNG sites lost. Whether this modulation at the genetic level increases or decreases the capacity of the virus to be neutralized by 2G12 remains to be elucidated. This would support our hypothesis that CQ could be considered as a therapeutic agent that does not only reduce viral load but which can also modify the gp120 envelope to induce a broader array of neutralizing Abs. Previous reports have indicated that alterations to PNG sites of the gp120 structure can provide for altered immune escape [3-5]. The PNG events on the gp120 molecule have been referred to as providing a "glycan shield", whereby the epitopes responsible for neutralization are protected. Modulating the gp120 envelope glycosylation patterns through treatment with CQ may have the benefit of broadening the Ab repertoire in treated individuals and hence providing better control of *in vivo *viral replication.

CQ has been shown to impair the formation of glycosylated epitopes on gp120 which are known to be involved with the binding of 2G12 [1,2]. The epitopes on the gp120 envelope that are involved with the 2G12 interaction are at amino acid positions 295, 332, 386, 392, 397 and 448 [23]. It is known that the binding sites on gp120 that interact with 2G12 and DC-SIGN are overlapping and encompass PNG events. Binding of cellular DC-SIGN can be reduced by the 2G12 Ab [29], although there have been reports demonstrating that 2G12 does not block the DC-SIGN interaction [30]. Our results with the CQ passaged virus show a loss of PNG sites at positions 332 and 397 of gp120, which have been shown to be an integral part of the 2G12 binding epitope. The loss of these amino acids may also explain the reduction in the DC-SIGN mediated transfer of the CQ passaged virus. Variation in the V1V2 and V3 regions have also been shown to be involved with altered DC-SIGN interactions [20], hence the genotypic alterations observed in the long-term culture may well be expected to alter the ability of the virus to be transferred to CD4^+ ^T-lymphocytes by cells expressing DC-SIGN. Our results with the C33A produced viruses indicate, however, that the decrease in DC-SIGN mediated viral transfer can also be exerted through single-cycle production of virus suggesting that CQ can affect the post-translational modification of the gp120 molecule. The similar infectivity phenotype of these viruses on CD4^+ ^T-lymphocytes alone suggests that the reduction in infectivity in the presence of Raji-DC-SIGN cells is mediated via the interaction with the DC-SIGN molecule.

The observed reduction of DC-SIGN mediated transfer could have implication for HIV-1 transmission. DC-SIGN has been implicated to play a role in the sexual transmission of HIV-1 and presumably other mucosal transmission routes, such as via breast-feeding [10,14,31-34]. The virus can interact with DC-SIGN and other C-type lectins expressed by DCs, which results in internalization of the virus. Maturation of the DCs results in migration to the lymph nodes where HIV-1 can be presented to a pool of CD4^+ ^T-lymphocytes and establish infection. Transmission of viruses from a CQ treated patient may therefore be more difficult to transmit via this route due to weaker DC-SIGN interactions. Although the reduction we observe in DC-SIGN mediated transfer of HIV-1 to CD4^+ ^lymphocytes is low any reduction in DC-SIGN mediated capture of virus at sites of exposure may have a significant repercussion on lowering rates of transmission, given the relative inefficiency of infection [35,36]. The effect of CQ on DC-SIGN binding *in vivo *remains to be determined, but if the DC-SIGN binding is indeed reduced than CQ treatment could be considered as a strategy to reduce transmission of HIV-1, again advocating for the use of the drug in specific cases where infection is more likely to occur.

## Conclusion

We have shown in this study that the effects exerted by CQ on reducing HIV-1 replication *in vitro *of both R5 and X4 viruses is exerted at the cellular level and that viruses produced via single round replications or via multiple passage are as infectious and replicate as efficiently as those produced in the absence of CQ. We have shown that HIV-1 passaged with CQ or produced in a single cycle production assay are less efficiently transferred to CD4^+ ^T-lymphocytes via DC-SIGN expressing cells than viruses produced in the absence of the drug. These results indicate that the effectiveness of CQ in reducing viral loads may have its effects exerted through multiple mechanisms. Additionally, we have identified that PNG patterns of the virus can alter when passaged in CQ indicating that *in vivo *the drug could be utilized as an agent to alter the immunogenic properties of gp120 in order to induce a broader range of neutralizing antibody responses and hence aide in the lowering of viral loads. The significance of these findings to the *in vivo *setting will be identified through the study of HIV-1 infected individuals treated with CQ.

## Methods

### Cells

Raji and Raji-DC-SIGN cells were cultured and utilized as previously described [10,37]. Peripheral Blood Mononuclear Cells (PBMCs) were isolated from three buffy coats from different HIV-1 uninfected donors by standard Ficol-Hypaque density centrifugation, pooled and frozen in multiple vials. After thawing, PBMCs were activated with phytohemagglutinin (2 μg/ml) and cultured in RPMI medium containing 10% FCS, penicillin (100 units/ml) and streptomycin (100 μg/ml) with recombinant interleukin-2 (100 units/ml). On day 3 the cells underwent CD8^+ ^cell depletion using CD8 immunomagnetic beads according to the manufacturers instructions and CD4^+ ^T-lymphocytes were cultured with IL-2 (100 units/ml). The human carcinoma cell line C33A was cultured in DMEM, with 10% FCS, penicillin (100 units/ml) and streptomycin (100 μg/ml).

### Generation of HIV-1 stocks

Replication competent HIV-1 stocks of JR-CSF (R5), LAI (X4) and 293.10 (R5X4) molecular cloned viruses (previously described in ref [38] were generated by passaging virus through isolated CD4^+ ^T-lymphocytes. Virus stocks were also produced by transfection of C33A cells with plasmid expressing the specific virus strain to be analyzed and using the assay described below. The CA-p24 levels in the culture supernatants were determined using a standard ELISA protocol. Virus stocks were generated either in the presence (100 μM) or absence of CQ.

### Long-term passage of HIV-1 in the presence and absence of CQ

The R5X4 dual-tropic molecular cloned virus (293.10) was the starting virus for the passage and has been previously described [38]. Fifteen ng/ml of CA-p24 was added to 10 × 10^6 ^CD4^+ ^T-lymphocytes in a volume of 5.0 ml RPMI medium with 100 μM CQ being added to the CQ passage culture flask. HIV-1 CA-p24 concentration was determined once a week with new virus being added to fresh CD4^+ ^T-lymphocytes. If the CA-p24 levels were too low then the CA-p24 was re-determined 3 days later with 15 ng/ml subsequently added to fresh cells. Remaining culture supernatants and cell pellets after each passage were stored at -80°C until required for analysis.

### TCID_50_/ml determination of generated viral stocks

TCID_50_/ml values were determined by limiting dilution of the viral stock on CD4^+ ^T-lymphocytes, as previously described [38]. In short the CD4^+ ^T-lymphocytes were plated at 2 × 10^5 ^cells/well in 96 well plates with 5 fold serial dilution of the virus. On day 7 the wells were scored for CA-p24 levels and the number of positive wells determined. These values were used to determine the TCID_50_/ml values for each virus. For the determination of the TCID_50_/ml for the C33A generated viruses and the viruses after prolonged culture, the input was standardized at 105 ng/ml CA-p24 and 10.5 ng/ml CA-p24, respectively.

### Replication curves of HIV-1 stocks

CD4^+ ^T-lymphocytes were plated at 1 × 10^5 ^cells/well in 96 well plates. One hundred TCID_50 _of virus stock was utilized with CQ being added either at 200 μM, 100 μM or 50 μm/well. For replication analysis of the C33A generated viruses and the viruses obtained from the prolonged CQ passage 1 ng/ml of CA-p24 was utilized as virus input. When analyzing viruses obtained from CQ cultures the level of CQ in the culture supernatant was compensated for in the control culture or TCID_50_/ml determination assay. CA-p24 values were determined using a standard ELISA assay for the culture supernatants obtained from the infection assay collected over time. All experiments were performed in triplicate with the standard means depict.

### Transfection of C33A cells with virus expressing plasmids

Transfection of C33A cells was performed with 10.0 μg of plasmid DNA expressing HIV-1 using the CaCl_2 _precipitation method. All plasmid DNA used was prepared using Qiagen kits. The DNA precipitate was split between two wells of C33A cells plated 24 hours earlier at 1.5 X10^6 ^cells/well in a 6 well tissue culture plate in DMEM medium either in the absence or presence of CQ (100 μM). The transfections were performed in a final concentration of 6 ml of DMEM, with penicillin (100 units/ml), streptomycin (100 μg/ml) and 10% FCS. The following day the cells were washed with PBS and fresh media was added, the viral stock was harvested on day 3 of culture with the viral CA-p24 levels determined by standard ELISA.

### DC-SIGN mediated HIV-1 transfer assay

The assay was performed as previously described [37]. The Raji and Raji-DC-SIGN cells were plated at a concentration of 2 × 10^4 ^cells/well in a 96 well format. Four hundred ng/ml of the appropriate virus was added to the Raji-DC-SIGN or Raji cells when studying the C33A produced virus stocks. For the CQ passaged viruses a set CA-p24 input of virus was utilized for each virus set (range 100 – 400 ng/ml). For the CQ passaged viruses the presence of CQ in the supernatant was compensated for in the control virus stock with an equal concentration of CQ added. After 2 hr incubation the culture was washed with PBS before addition of CD4^+ ^T-lymphocytes at a concentration of 1 × 10^5 ^cells/well. CA-p24 values were determined on day 7 using a standard ELISA protocol and all experiments were performed in triplicate.

### Sequencing and sequence analysis

HIV-1 RNA was isolated from culture supernatant according to the method of Boom [39]. Viral RNA was converted to cDNA and then subjected to a nested PCR to amplify a fragment covering the V1V2 to C4 region of the gp120 gene. DNA sequence alignments were performed manually. Positions containing an alignment gap were included for the pair-wise sequence analysis. Phylogenetic analysis of the amplified region was performed with the neighborhood-joining (N-J) analysis of MEGA [40]. The PNG sites were analyzed using the program available at the HIV-1 sequence database [41].

### Statistics

All statistical comparisons were performed using ANOVA with P < 0.01 being considered as statistically significant.

## Abbreviations

Ab, antibody; CQ, chloroquine; DC, dendritic cell; DC-SIGN, DC-pecific ICAM3 grabbing non-intergrin; ECD, extra-cellular domain; HCQ, hydroxychloroquine; R5, CCR5 coreceptor using HIV-1 isolate; R5X4, CCR5 and CXCR4 coreceptor using HIV-1 isolate; X4, CXCR4 using HIV-1 isolate; PNG; potential N-linked glycosylation.

## Competing interests

The author(s) declare that they have no competing interests.
